# HIV-1 Tat Interacts with and Regulates the Localization and Processing of Amyloid Precursor Protein

**DOI:** 10.1371/journal.pone.0077972

**Published:** 2013-11-29

**Authors:** Jiyoung Kim, Jee-Hyun Yoon, Yeon-Soo Kim

**Affiliations:** 1 Indang Institute of Molecular Biology, Inje University, Jung-Gu, Seoul, Republic of Korea; 2 Department of Smart Foods and Drugs, Inje University, Jung-Gu, Seoul, Republic of Korea; University of South Carolina School of Medicine, United States of America

## Abstract

HIV-1 Tat protein plays various roles in virus proliferation and in the regulation of numerous host cell functions. Accumulating evidence suggests that HIV-1 Tat also plays an important role in HIV-associated neurocognitive disorders (HAND) by disrupting intracellular communication. Amyloid beta (Aβ) is generated from amyloid precursor protein (APP) and accumulates in the senile plaques of Alzheimer's disease patients. This study demonstrates that Tat interacts with APP both *in vitro* and *in vivo*, and increases the level of Aβ42 by recruiting APP into lipid rafts. Co-localization of Tat with APP in the cytosol was observed in U-87 MG cells that expressed high levels of Tat, and redistribution of APP into lipid rafts, a site of increased β- and γ-secretase activity, was demonstrated by discontinuous sucrose density gradient ultracentrifugation in the presence of Tat. Furthermore, Tat enhanced the cleavage of APP by β-secretase *in vitro*, resulting in 5.5-fold higher levels of Aβ42. This was consistent with increased levels of β-C-terminal fragment (β-CTF) and reduced levels of α-CTF. Moreover, stereotaxic injection of a lentiviral Tat expression construct into the hippocampus of APP/presenilin-1 (PS1) transgenic mice resulted in increased Tat-mediated production and processing of Aβ *in vivo*. Increased levels of Aβ42, as well as an increase in the number and size of Aβ plaques, were observed in the hippocampus following injection of Tat virus compared with mock virus. These results suggest that HIV-1 Tat may contribute to HAND by interacting with and modifying APP processing, thereby increasing Aβ production.

## Introduction

The human immunodeficiency virus type 1 (HIV-1) Tat is an important regulator of viral transcription. The primary role of Tat is transactivation of the HIV-1 long-terminal repeat promoter, which is essential for viral replication [1]. In addition, HIV-1 Tat is involved in various cellular processes including the regulation of translation [2], [3], induction of angiogenesis [4], modulation of cytokine expression [5], and activation of cellular signaling pathways [6]. The HIV-1 Tat protein is secreted from virus-infected cells in the brains of AIDS patients [7]. It is taken up by astrocytes and neurons [8] and may contribute to brain damage by increasing the levels of intracellular calcium and reactive oxygen species in neurons, resulting in neuronal apoptosis [9], [10] and the subsequent production of chemokines by astrocytes, which recruit monocytes to the site of inflammation [11] and/or disrupt blood-brain barrier integrity [12].

Senile plaques are lesions frequently found in the brains of Alzheimer's disease (AD) patients. Senile plaques are composed of extracellular deposits of a small peptide (39–42 amino acids in length) termed amyloid-β (Aβ). Aβ is generated from a large type I transmembrane protein, amyloid precursor protein (APP) [13], [14], via sequential cleavage by α- or β- and γ-secretases [15]. Most newly synthesized APP is cleaved by α-secretase [16], resulting in a large N-terminal fragment called sAPPα and a short α-C-terminal fragment (α-CTF). This process is known as the non-amyloidogenic pathway. Less frequently, APP is cleaved by β-secretase [17], generating sAPPβ and β-CTF. This pathway is known as the amyloidogenic pathway. Both α- and β-CTF are subsequently cleaved by γ-secretase [18] to yield p3 and Aβ, respectively. Cleavage of CTF by γ-secretase also generates a cytoplasmic peptide termed the APP intracellular domain [15].

APP is produced in the endoplasmic reticulum and transported through the trans-Golgi network to the plasma membrane [19]. A portion of APP is internalized by endocytosis, which is important for amyloidogenic processing and Aβ production [20]. Proper subcellular localization and trafficking of APP is an important factor in the generation of Aβ. Many cellular proteins, including cytoplasmic adapter proteins such as Fe65 [21], X11 [22], JIP1 [23], Dab1 [24], and sorting Nexin 17 [25], interact with APP and modulate its trafficking and processing, thereby increasing or decreasing the levels of Aβ. Other proteins that interact with APP and modulate its trafficking include sorting protein-related receptor sorLA [26], ubiquilin1 [27], which plays a central role in the regulation of proteasomal degradation [28], and low-density lipoprotein receptor-related protein (LRP) [29], which is involved in endocytosis and intracellular signaling [30].

Accumulating evidence suggests that the HIV-1 Tat protein plays an important role in the processing and accumulation of Aβ in the brains of AIDS patients. First, Rempel and Pulliam (23) showed that HIV-1 Tat interacts with and inhibits neprilysin, the major Aβ-degrading enzyme in the brain. Levels of Aβ are also significantly increased in postmortem brain samples from patients infected with HIV-1 [31]. Second, addition of recombinant Tat inhibits the uptake of Aβ by primary mouse microglial cells, suggesting that HIV-1 Tat regulates the level of Aβ by inhibiting microglial phagocytosis [32]. Third, the induction of Tat in astrocytes increases neuronal damage, tau phosphorylation, and Aβ plaque formation in APP/presenilin-1(PS1) transgenic mice [33], suggesting an important role for HIV-1 Tat in the development of HIV-1-associated neurocognitive disorders (HAND).

Here, we present data suggesting that HIV-1 Tat contributes to the increased levels of Aβ in cultured astroglial cells and in the brains of APP/PS1 mice by interacting with APP and regulating its trafficking and processing. These findings provide critical new insights into the functions of HIV-1 Tat.

## Results

### HIV-1 Tat Interacts with APP both *In Vitro* and *In Vivo*

**Figure 1:**
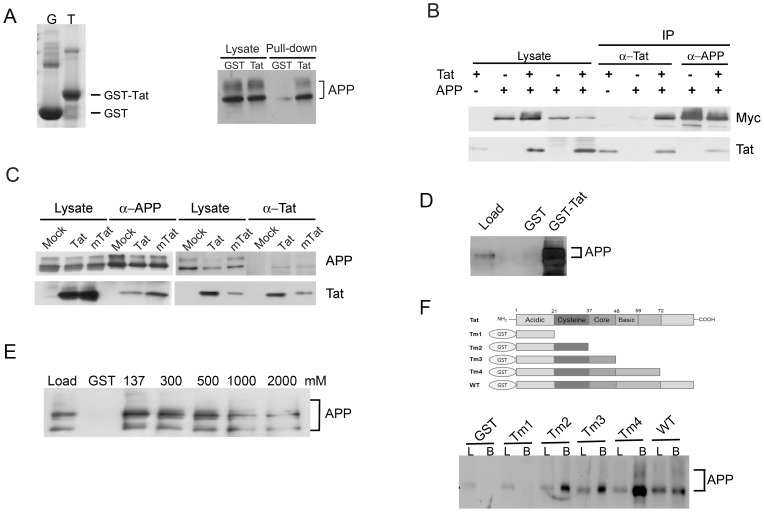
HIV-1 Tat interacts with APP both *in vitro* and *in vivo*. (*A*) GST and GST-Tat were purified on glutathione-Sepharose beads. The beads were boiled to elute the bound proteins, which were then run on a 12% SDS-PAGE gel and stained with Coomassie brilliant blue (left panel). GST pulldown assay with SK-N-MC neuroblastoma cell lysates shows a strong interaction between APP and GST-Tat (right panel). SK-N-MC neuroblastoma cell extracts incubated with GST- or GST-Tat-coated beads for 3 hours. The beads were washed three times with PBS and the eluted proteins were analyzed by western blotting with an anti-APP antibody (22C11). (*B*) Coimmunoprecipitation of Tat and APP in HEK 293FT cells transfected with Tat and/or Myc-tagged APP695 vectors. Proteins were precipitated with anti-Tat or anti-APP (6E10) antibodies and immunoblotted with anti-Tat or anti-Myc antibodies. (*C*) Coimmunoprecipitation of U-87 MG cell lysates transduced with mock, Lenti-Tat, or Lenti-mTat virus. APP was precipitated with an APP antibody (6E10), and the precipitate was analyzed by SDS-PAGE followed by Western blotting with anti-APP (22C11) or anti-Tat antibodies. Reciprocally, Tat was precipitated with anti-Tat antibody, and the precipitate was analyzed by SDS-PAGE followed by Western blotting with anti-APP (A8717) or anti-Tat antibody. (*D*) Purified recombinant APP interacts with GST-Tat. Purified recombinant APP (500 ng) was incubated with GST- or GST-Tat-coated beads and the eluted proteins were analyzed by western blotting with an APP antibody. A large amount of recombinant APP bound to the GST-Tat beads. (*E*) Tat interacts strongly with APP. SK-N-MC neurobalstoma cell lysates were incubated with GST, GST-Tat, or GST-Tat beads, and washed three times in buffer containing 137, 200, 300, 400 or 500 mM NaCl. APP remained associated with GST-Tat under high-salt conditions. (*F*) The cysteine-rich domain of Tat is important for association with APP. Deletion mutants were produced as GST-fusion proteins and subjected to GST-pulldown assays with SK-N-MC cell lysates. L, load; B; bound.

To evaluate the interaction between Tat and APP, a recombinant Tat protein fused to glutathione S-transferase (GST) was produced in *Escherichia coli* (Figure 1*A*). SK-N-MC neuroblastoma cell lysates were applied to GST-Tat beads and the bound proteins were eluted and probed by Western blotting with an antibody against APP. The results consistently showed a strong interaction between APP and GST-Tat, but not with GST alone (Figure 1*A*). Both the newly synthesized (lower band) and post-translationally modified forms of APP (upper band) bound to GST-Tat (Figure 1*A*).

To confirm the association of Tat with APP, expression vectors for Tat- and Myc-tagged human APP695 were cotransfected into HEK 293T cells. Tat and APP were immunoprecipitated with anti-Tat or anti-APP antibodies, respectively, and the precipitated proteins were examined by Western blotting with antibodies to Myc or Tat. When Tat protein was precipitated, APP was co-precipitated. Reciprocally, when APP was precipitated, Tat was co-precipitated (Figure 1*B*). These results suggest a strong interaction between APP and Tat. To discount the possibility that the interaction between Tat and APP was an artifact resulting from protein overexpression, human glioblastoma U-87 MG cells were transduced with a recombinant lentivirus containing the Tat expression construct under the control of the CMV promoter (Lenti-Tat). Immunoprecipitation with an anti-APP antibody, 6E10, followed by Western blotting with an anti-APP antibody, 22C11, or an anti-Tat antibody confirmed that APP interacted with Tat in U-87 MG cells (Figure 1*C*). In immunoprecipitations using anti-Tat antibody, APP was co-precipitated with Tat (Figure 1*C*).

Of central importance to this study is the question of whether the association between APP and Tat is direct, or whether they interact indirectly via a cellular cofactor, such as P-TEFb, which interacts with Tat and is involved in Tat transactivation. To evaluate this, an expression construct for a mutant Tat (mTat), in which lysine 41 is replaced by glutamate (TatK41E), thereby rendering mTat deficient in transactivation [34], was expressed in U-87 MG cells, and its interaction with APP was investigated. A recombinant lentivirus containing a mock expression vector was also produced and used as a control. mTat did not exhibit transactivation activity when the protein was expressed in Magi (HeLa-CD4-LTR-β-gal) cells, which express β-galactosidase under the control of the HIV-1 LTR promoter, when these cells were subsequently stained with X-gal ( Figure *S*1*A*). However, the protein exhibited a strong interaction with APP (Figure 1*C*), indicating that the interaction between Tat and APP does not require Tat-dependent expression of a mediator protein.

We also examined the possibility that the association between Tat and APP is mediated by other cellular factors such as RNA. To rule out any such effects, we incubated purified recombinant APP proteins (Figure *S*1*B*) with beads coated with GST or GST-Tat. The association between Tat and APP was then examined by western blotting with an antibody against APP. A large amount of APP bound to GST-Tat but not to GST alone, suggesting that Tat directly interacts with APP (Figure 1*D*).

To examine the affinity of Tat for APP, SK-N-MC cell extracts were incubated with GST- or GST-Tat-coated beads. The GST-Tat beads were washed three times with a buffer containing 137 mM, 300 mM, 500 mM, 1M or 2M NaCl, and samples from each wash were analyzed by western blotting with an anti-APP antibody (Figure 1*E*). A large amount of APP remained bound to GST-Tat after washing with 500 mM NaCl, indicating that the interaction between APP and Tat was stable at medium salt concentrations. Significant amount of APP remained bound to GST-Tat after washing with buffer containing 1M NaCl, while most of APP dissociated after washing with buffer containing 2M NaCl (Figure 1*E*). This suggests that the affinity of App for Tat is moderate-to-strong.

Tat is composed of five different functional domains that play important roles in transactivation (cysteine-rich domain), nuclear localization, and RNA binding (basic domain) (Figure 1*D*). To determine which region of Tat is involved in interaction with APP, we constructed serial C-terminal deletion mutants of Tat in the form of GST-fusion proteins. Deletion mutants (Figure *S*1*C*) were subjected to GST-pull-down assays using SK-N-MC cell lysates. As shown in Figure 1*D*, APP binds to the Tat-containing acidic and cysteine-rich domain but not to the acidic domain alone, suggesting that the cysteine-rich domain of Tat is important for APP binding (Figure 1*F*).

Based on the results of the experiment showing that the cysteine-rich domain of Tat is required to APP binding, we tried to identify a mutant Tat protein that does not bind to APP. Three Tat mutants harboring a mutation in the cysteine-rich domain were generated and expressed as GST-fusion proteins: TatC22G (in which the cysteine 22 was replaced with glycine), TatH33A (in which the histidine 33was replaced with alanine), and TatΔC34Q35 (in which cysteine 34 and glutamine 35 were deleted) (Figure *S*1*D*). The interaction between the mutant-Tat proteins and APP was examined in a GST pull-down assay using SK-N-MC cell extracts. All three mutants bound to APP (Figure *S*1*E*).

### HIV-1 Tat Co-localizes with APP and Promotes Lipid Rafts Localization of APP

**Figure 2:**
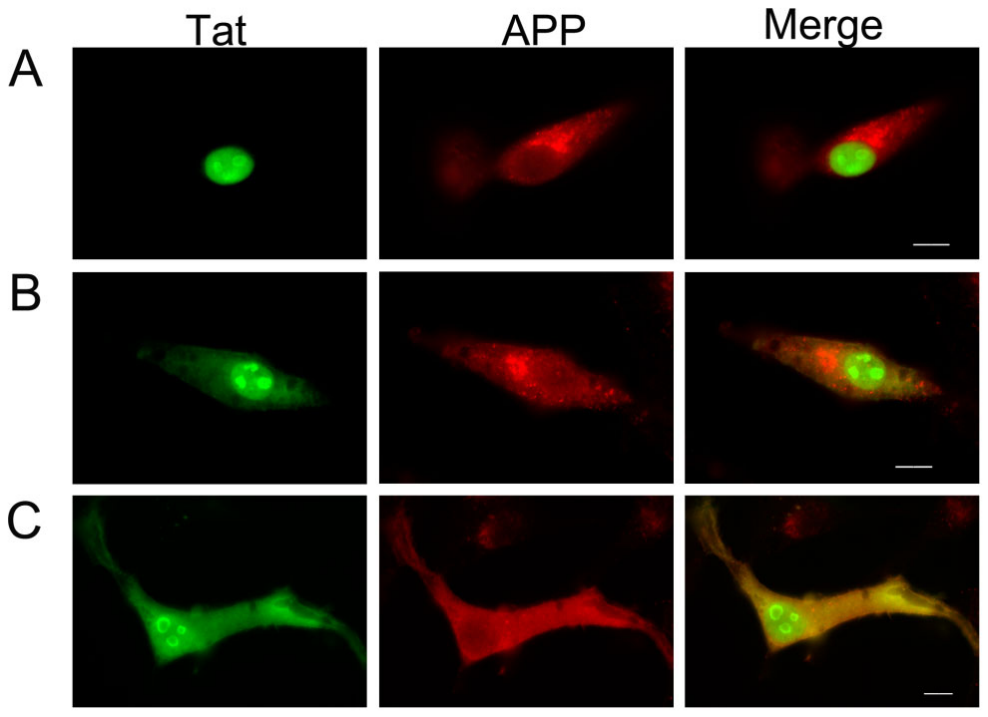
HIV-1 Tat colocalizes with APP in U-87 MG cells. Fluorescence microscopy images of Tat-transfected U-87 MG cells immunostained with anti-Tat and anti-APP (A8717) antibodies are shown. U-87 MG cells were transfected with the wild-type Tat construct and incubated for 16 h. The cells were fixed and stained with anti-Tat or anti-APP antibodies followed by FITC-conjugated anti-mouse or rhodamine-conjugated anti-rabbit antibodies, respectively. Nuclear (*A*) and nuclear plus cytosolic (*B* and *C*) localization of Tat is shown. Scale bar = 10 µm.

To further examine the interaction between Tat and APP, U-87 MG human glioblastoma cells were transfected with an expression construct for Tat and immunostained with anti-Tat and an anti-APP antibody (A8717) that recognizes the C-terminus of APP. Although Tat is a nuclear protein, a significant amount is found in the cytosol of human brain bizarre astrocytes, primary astrocyte cultures infected with HIV, U-87 MG glioblastoma cells [35], and astrocytes derived from the brains of AIDS encephalopathy patients [36], [37]. Consistent with these results, Tat was observed in both the nucleus and the cytosol of many transfected U-87 MG cells (Figure 2*B and C*, *Figure S2A*). Co-localization of Tat and APP was also observed in these cells. Interestingly, the distribution of APP appeared to be dependent on the level of Tat in the cytosol. In cells in which only nuclear Tat was detected, APP was primarily localized in foci in the perinuclear region (Figure 2*A*). These foci disappeared as the levels of cytosolic Tat protein increased (Figure 2*B*). In cells expressing high levels of Tat in the cytosol, APP was dispersed throughout the cytosol, and no foci were evident (Figure 2*C*). Expressed mTat showed the same expression pattern as wild-type Tat (Figure *S*2*B*).

**Figure 3:**
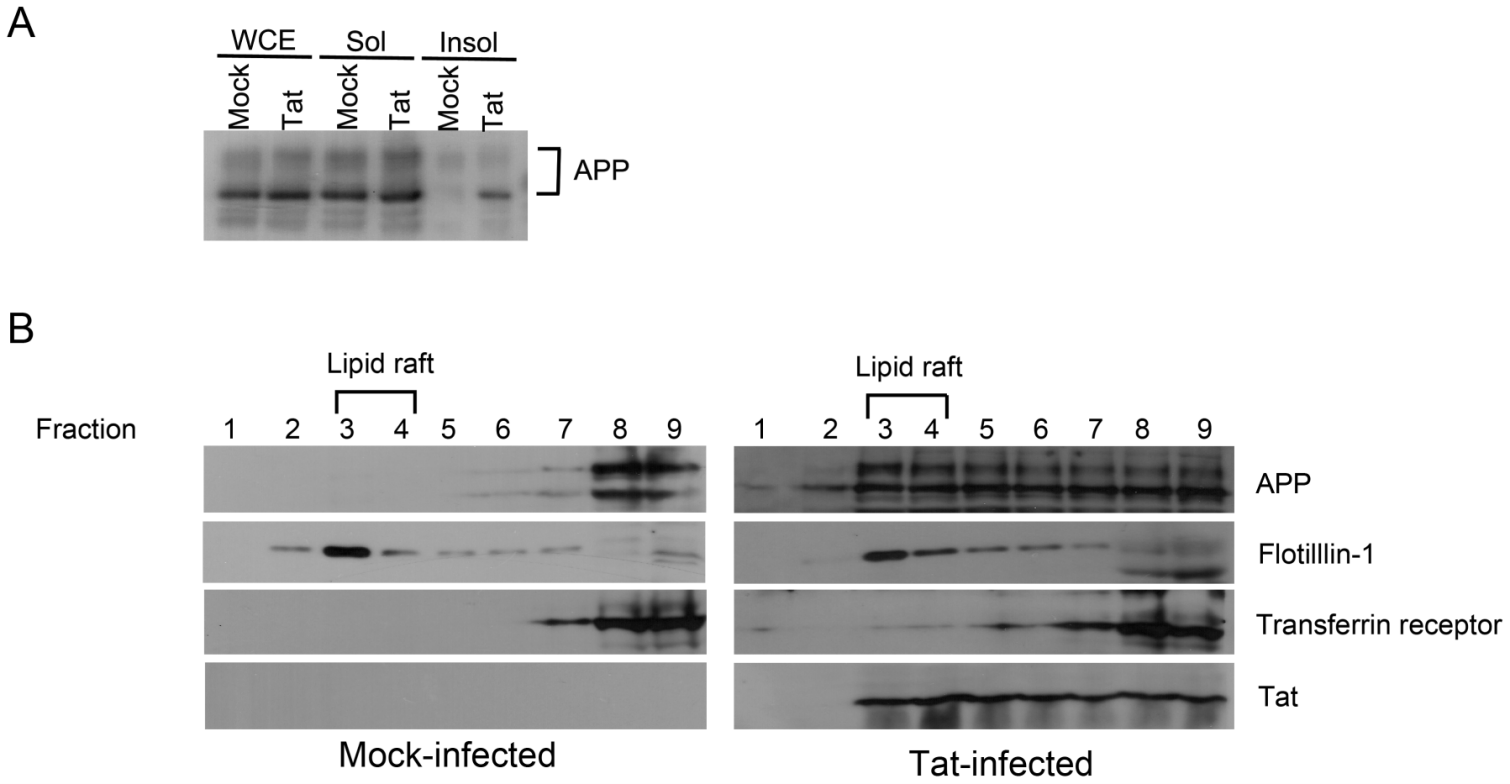
HIV-1 Tat promotes localization of APP to lipid rafts. (*A*) The amounts of Triton X-100-resistant APP increased in Lenti-Tat infected U-87 MG cells. U-87 MG cells were transduced with mock virus or with Lenti-Tat virus, and incubated for 20 days. Cells were harvested and lysed in the presence of 1% Triton X-100 and the soluble and insoluble fractions were separated by centrifugation. WCE; whole cell extracts, Sol; soluble fraction, Insol; insoluble fraction. (*B*) U-87 MG cells were transduced with mock virus or Lenti-Tat virus and incubated for 14 days. Cells were harvested and lysed in the presence of 1% Triton X-100 and subjected to 5% and 35% discontinuous sucrose density gradient ultracentrifugation. Fractions of 0.5 ml were harvested from the top to the bottom and analyzed by Western blotting for APP, flotillin-1, transferrin receptor and Tat. The majority of APP was found in the non-raft fractions in mock –infected cells (Left panel) while large amount of APP was moved to lipid raft fractions in Lenti-Tat infected cells (Right panel).

APP solubility in the presence of Tat was examined by solubilizing the mock- or Lenti-Tat-infected U-87 MG cells in a buffer containing 1% Triton X-100. The Triton X-100 soluble and insoluble fractions were separated by centrifugation and the amount of APP was examined by western blotting with APP antibody (22C11). Most of the APP was found in the Triton X-100 soluble fractions as already known [38] in mock-virus infected cells (Figure 3*A*). However, large amount of APP was resistant to Triton X-100 and remained in the insoluble fraction (Figure 3*A*) in Lent-Tat-infected cells, suggesting that Tat changed the localization of APP. In an effort to understand how Tat expression alters APP solubility, the presence of APP in lipid rafts was examined. Growing evidence suggests that both β- and γ-secretases are enriched in lipid rafts, and that the amyloidogenic processing of APP by secretases takes place in these membrane domains [39], [40], [41], [42]. In fact, targeting β-secretase to lipid rafts up-regulates β-site processing of APP, resulting in increased levels of Aβ [39].

To examine the distribution of APP in lipid rafts, cell extracts were prepared from mock- or Tat-virus-infected U-87 MG cells using 1% Triton X-100. The extracts were then fractionated on 5% and 35% discontinuous sucrose density gradients. The gradient fractions were then analyzed by Western blotting with anti-APP, anti-Tat, anti-transferrin receptor and an antibody against flotillin-1 [43], a marker protein for lipid rafts (Figure 3*B*). Flotillin-1 was enriched in lipid raft fractions 3 and 4 at the interface between the 5% and 35% sucrose layers. The transferrin receptor, a non-lipid raft protein, was enriched in fractions 8 and 9, suggesting that the lipid rafts were separated efficiently (Figure 3*B*). Although most Tat was present in the heavy, non-raft cellular fractions, a significant amount was also found in the lipid raft fractions, as was a large amount of APP (Figure 3*B*). This is consistent with the hypothesis that APP is recruited to lipid rafts by Tat. In mock virus-infected cells, the majority of APP was found in the heavy, non-raft fractions (Figure 3*B*). Similar amounts of APP were found in the lipid raft fractions of Lenti-mTat-infected cells compared with cells expressing Tat (Figure *S*3).

### HIV-1 Tat Increases the Level of Aβ and β-CTF in U-87 MG Cells

**Figure 4:**
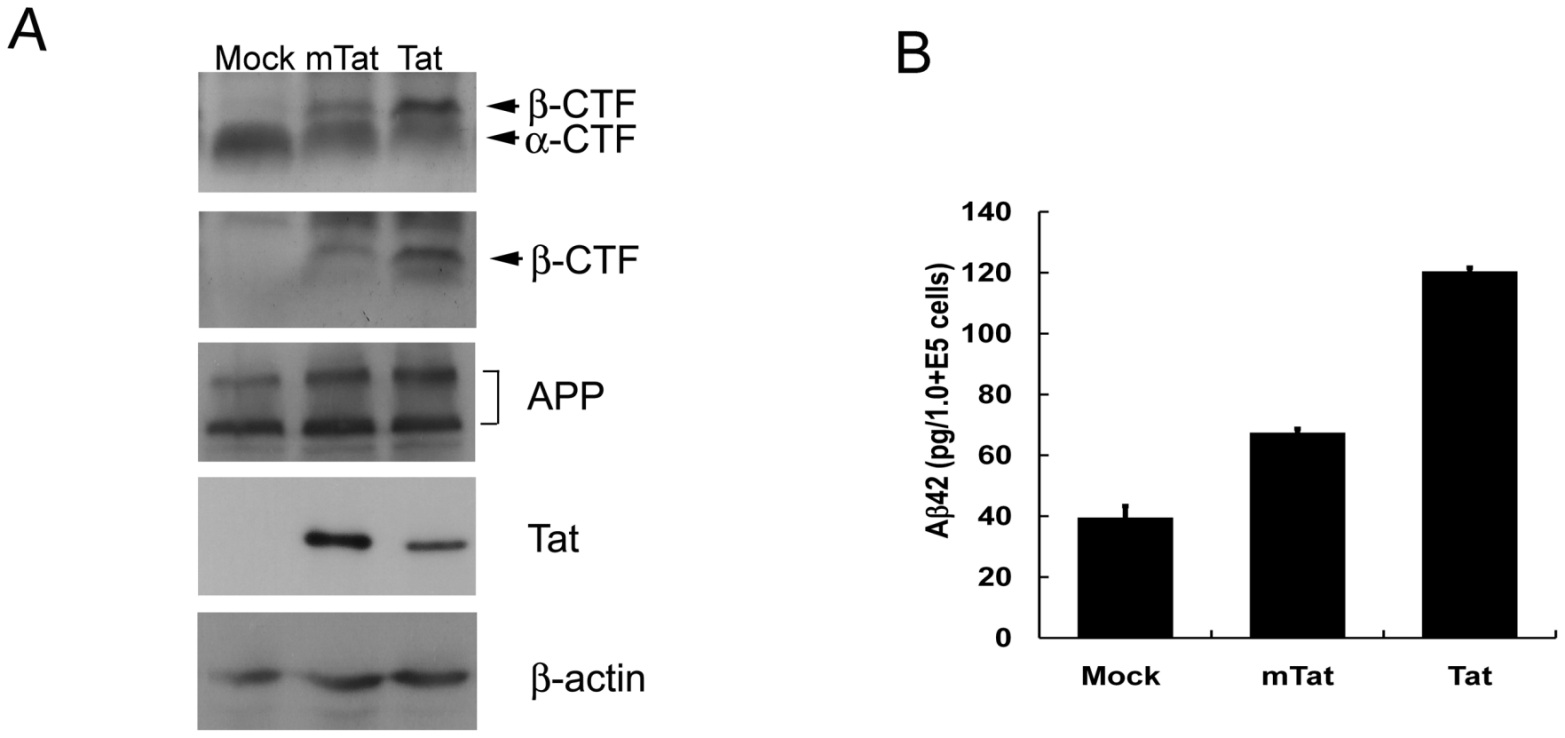
HIV-1 Tat alters the processing of APP and increases the levels of β-CTF and Aβ42. (*A*) Level of β-CFT was increased in Lenti-Tat, or Lenti-mTat infected cells while the amount of total APP was not changed. U-87 MG cells were transduced with mock, Lenti-Tat, or Lenti-mTat virus and incubated for 14 days. The cell number was counted, and equal numbers of cells from each sample were analyzed in 16.5% Tris-Tricine gels to detect CTF or 8% SDS-PAGE for the detection of APP and β-actin. Both α- and β-CTF were detected with an anti-APP C-terminal antibody (A8717, top panel), and β-CTF was detected with 6E10 (upper middle panel). The total amounts of APP (22C11, lower middle panel) and β-actin (bottom panel) were not changed by the expression of Tat or mutant Tat protein. (*B*) Aβ42 level was increased in Lenti-Tat and Lenti-mTat infected U-87 MG cells. Average of Aβ42 concentration for 9 days was calculated from individual Aβ42 concentration. The Aβ42 concentrations in Lenti-Tat- or Lenti-mTat-infected cells were increased by 5.58±0.83 (mean ± SE, P<0.005)-fold or 3.64±0.38 (mean ± SE, P<0.005)-fold, respectively, compared with mock-infected cells. (*C*) Treatment with the neprilysin inhibitor thiorphan further increased the level of Aβ42 in the presence of Tat. Mock or Lenti-Tat-infected U-87 MG cells were cultured for 3 days in the absence or presence of 10 µM thiorphan. Culture supernatant was harvested from each cell culture and analyzed by Aβ42 ELISA. Error bars represent the mean ± SD. *P<0.05, **P<0.001.

To determine whether the interaction with and putative relocalization by Tat affects the proteolytic processing of APP, U-87 MG cells transduced with Lenti-Tat, Lenti-mTat, or mock virus were incubated for 14 days, and the level of α- or β-CTF was evaluated by Western blotting. The level of APP was not affected by the expression of either wild-type Tat or mTat protein (Figure 4*A*). However, the amount of β-CTF was markedly increased in Tat-infected cells, whereas the level of α-CTF appeared to be somewhat decreased compared with that in mock-infected cells (Figure 4*A*, top panel). The increase in β-CTF was confirmed by immunostaining with an antibody that recognizes amino acids 1–17 of Aβ (Figure 4*A*, upper middle panel). Despite its elevated expression level, mTat induced a relatively small level [21]of β-CTF compared with wild-type Tat (Figure 4*A*). This suggests that Tat recruits APP into lipid rafts to facilitate the processing of APP by β-secretase.

Next, the level of Aβ in Tat-expressing cells was examined by ELISA. Mock, Tat, or mTat-infected U-87 MG cells were cultured for 9 days. Conditioned media were harvested every 3 days, and the levels of Aβ42 were examined by ELISA. As already known [7], Tat induced toxicity in neuroblastoma SH-SY5Y cells (Figure *S*4) and U-87 MG cells (Figure *S*5*A*) resulting in retarded growth while mTat showed attenuated cytotoxicity in SH-SY5Y and U-87 MG cells (Figure *S*4 and *S*5*A*). Due to the vastly different growth rates of the cell types, the cell number was counted at each harvest (Figure *S*5*A*) and used to calculate the Aβ42 concentration. The concentration is given as the amount of Aβ42 produced by 1×10^5^ transduced U-87 MG cells (Figure *S*5*C*). Although the concentration of Aβ42 varied depending on the culture period (Figure *S*5*C*), Lenti-Tat-infected cells showed a 5.58±0.83 (mean ± SE)-fold increase in Aβ42 levels compared with mock-infected cells (*P*<0.005). The concentration of Aβ42 in Lenti-mTat-infected cells was 3.64±0.38 (mean ± SE)-fold higher than that in mock-infected cells (P<0.005) (Figure 4*B*). The moderate increase in Aβ42 levels in mTat-infected cells was consistent with the relatively small increase in β-CTF (Figure 4*A*).

### Aβ42 Levels are Increased in APP/PS1 Mice

**Figure 5:**
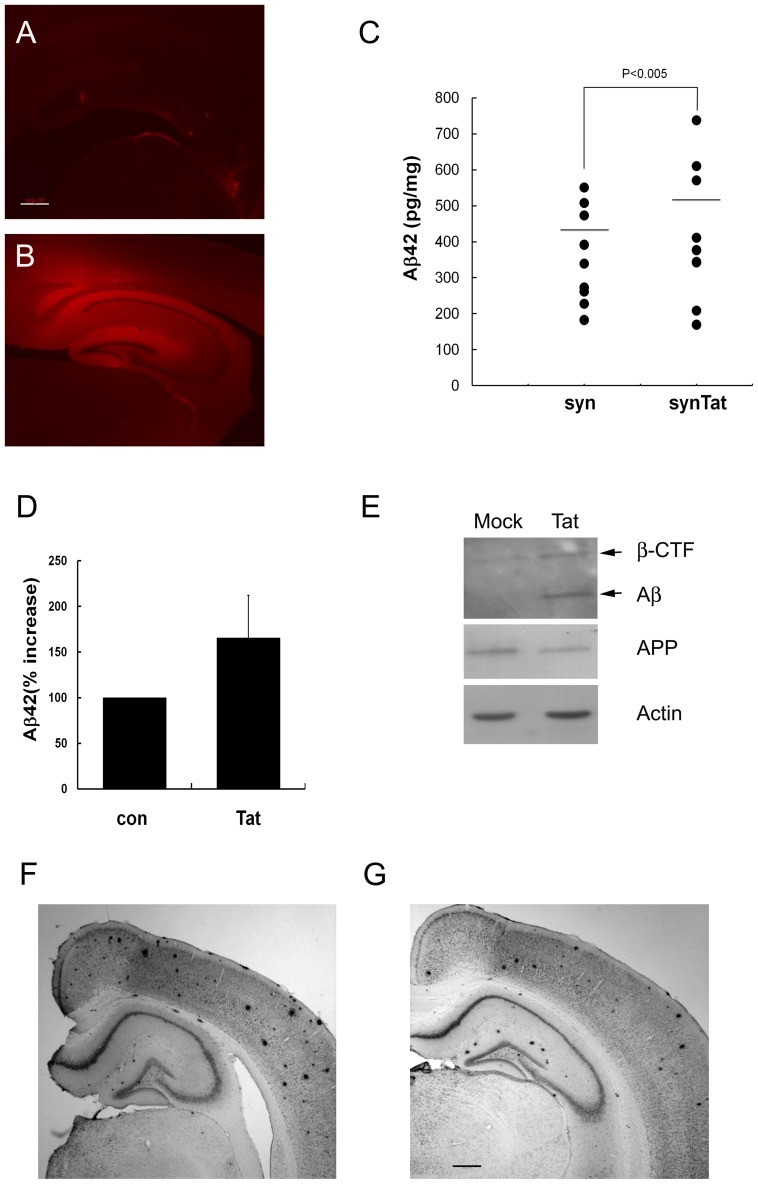
HIV-1 Tat increases Aβ levels and the number and size of amyloid plaques in the APP/PS1 mouse hippocampus. Mock (*A*) or syn-Tat (*B*) was injected stereotaxically into APP/PS1 mouse hippocampi. At 2 months after injection, half of the brain was frozen-sectioned and stained with anti-Tat antibody, and then visualized under an inverted phase fluorescence microscope. Scale bar = 50 µm. (*C*) Mouse hippocampi (n = 9) injected with mock (syn) or Tat (syn-Tat) were dissected, homogenized 4 months after injection, and Aβ42 levels in the hippocampus extracts were examined by ELISA. The Aβ42 concentration for each mouse is shown on the y-axis, and the injected virus is indicated on the x-axis. (*D*) Equal volumes of syn-Tat and Lenti-Tat were mixed and injected unilaterally into the mouse hippocampus (n = 5). After 4 months, the hippocampus was dissected from mock (con)- and mixed Tat (Tat)-virus-injected hemispheres, and Aβ42 levels were investigated by ELISA. Aβ42 levels were 1.6-fold higher in the Tat-expressing hemisphere of mouse hippocampi (P<0.001). (*E*) Representative Western blots for the detection of Aβ. Twenty micrograms of mouse hippocampus extract were separated on 16.5% Tris-Tricine PAGE gels followed by Western blotting with anti-APP (6E10) antibody. Five micrograms of hippocampus extract were used for Western blotting for APP and actin. (*F*, *G*) Representative photographs of the Aβ plaque burden in APP/PS1 mice are shown. Brain sections of APP/PS1 mice injected with mock (*F*) or Lenti-Tat (*G*) virus were stained with anti-Aβ antibody (6E10). Scale bar = 50 µm.

The effect of HIV-1 Tat on the processing of APP was examined using the APP/PS1 [44] transgenic mouse line. Lentiviral vectors containing the Tat expression construct under the control of the synapsin promoter (syn-Tat) [45] were injected stereotaxically into the hippocampi of 2-month-old APP/PS1 mice, and the expression of Tat was examined by immunostaining with an anti-Tat antibody (Figure 5*A and B*). Tat was observed throughout the hippocampus, the dentate gyrus, and the CA1 and CA3 pyramidal neurons (Figure *S*6*A*) at 2 months after injection. Co-staining of brain sections with anti-Tat antibody and an antibody against the neuronal marker microtubule-associated protein-2 (MAP2) [46] indicated that neuronal cells were the primary cell type transduced by the viral vector (Figure *S*6*B*). Mock-injected brains were not immunoreactive with the anti-Tat antibody (Figure 5*A*).

Groups of nine mice (2 months of age) were used for bilateral injection of syn-Tat or mock virus. Four months after injection, the mice were sacrificed and the hippocampus was dissected from one half of the coronal brain section to assess Aβ42 levels. The other half was frozen and sectioned for Aβ plaque staining. Although the level of Aβ in the Aβ42 mice fluctuated, the syn-Tat-injected mice showed a 20% increase in Aβ42 compared with mock-injected mice (P<0.005; Figure 5*C*).

To minimize the variation in Aβ levels between individual mice, Tat-expressing virus was injected unilaterally into the hippocampi of five mice, and the levels of Aβ42 in the hemispheres were compared. In this experiment, equal volumes of syn-Tat and Lenti-Tat viruses were mixed to increase Tat expression in the mouse hippocampus. In the presence of high levels of Tat expression, the level of Aβ42 increased 1.6-fold (*P*<0.001; Figure 5*D*). This increase was confirmed by Western blotting, which showed a large increase in β-CTF and Aβ compared with that in mock virus-injected mice (Figure 5*E*). The second half of the syn-Tat-injected mouse brains and the brains of mixed virus-injected mice were coronally sectioned and stained with anti-Aβ antibody (6E10). Consistent with the level of Aβ42 detected by ELISA, the number and size of the Aβ plaques in the mouse hippocampus were variable. However, the size and number of Aβ plaques in the Tat-virus-injected hippocampi were increased compared with the control side in brains that showed a similar number of plaques in the cortical region (Figure 5*F and G*). Note that the viruses were not injected into the cortex, and that the number and size of Aβ plaques in the cortex were not affected by Tat expression.

The plaques were counted using Axiovision software (Carl Zeiss), and the diameter of the plaques was measured to evaluate whether there was an increase in the number and/or size of Aβ plaques. The number of plaques increased by 1.26- and 1.9-fold in syn-Tat- or mixed virus-injected mouse hippocampi, respectively, compared with mock-injected or non-injected mouse hippocampi. The mean plaque size was 1.1- and 1.45-fold larger for syn-Tat- or mixed virus-injected hippocampi, respectively, than that in the control (Table 1).

**Table 1:** Number and mean plaque size in mock- or Tat-virus-injected APP/PS1 mouse hippocampi.

Virus	Mixed Tat		Syn-Tat	
	Control	Tat	Control	Tat
Number of sections	5	5	7	7
Number of plaques	45	86	68	86
Plaque number/brain slice	9	17.5	9.71	12.28
Mean Plaque size (µM)	24.919±4.46	36.151±3.37	40.587±8.92	45±5.64
% Increase		145.0734		110.893

Lentiviruses containing an expression construct for mock or Tat were injected stereotaxically into the hippocampus of APP/PS1 mice. After 4 months, sections of brain were stained with Aβ antibody and the number and size of plaques were determined using Axiovision software (Carl Zeiss). Results represent the mean ± s.d.

## Discussion

This study demonstrates that HIV-1 Tat, a regulator of viral transcription, directly interacts with APP and modulates the trafficking and processing of the protein, resulting in increased Aβ production. HIV-positive patients exhibit HIV-1-associated neurological disorders (HAND) characterized by cognitive, behavioral and motor dysfunction. Although the mechanism that mediates the onset of HAND in AIDS patients is unknown, it is believed that secreted viral proteins as well as chemokines and cytokines produced by activated macrophages, astrocytes, and microglia damage neuronal cells, play a role resulting in the loss of synaptic integrity and function. Some HIV-positive patients exhibit the HAND phenotype with an increase in deposition of Aβ [47], [48], [49], [50] or an increased level of phosphorylated tau protein [51], [52], [53]. Since the development of highly active anti-retroviral therapy (HAART), the morbidity of HIV-1-infected individuals has significantly reduced [54], while the prevalence of HAND appears to be increasing among AIDS patients. In addition, the clinical presentation of HAND has changed from a rapidly progressive HIV-1-associated dementia (HAD) to a mild neurodegenerative process involving neuronal cells that are not usually affected by acute HAD. Accumulating evidence shows that the deposition of Aβ and Tau increases in post-HAART patients [52], [55], suggesting that HAART treatment may increase the incidence of Alzheimer's disease. The evidence presented in this study demonstrates direct interaction (Figure 1), relocalization (Figure 2 and 3), and modification of APP processing (Figure 4 and 5) by Tat, resulting in increased levels of Aβ42 (Figure 4 and 5) in astrocytes (Figure 4) and in the APP/PS1 mouse brain (Figure 5). These results suggest that HIV-1 Tat may play a role in the development of HAND in post-HAART patients by promoting the cleavage of APP.

Accumulating data suggest that the HIV-1 Tat protein increases the level of Aβ by inhibiting the Aβ-degrading enzyme neprilysin [31], inhibiting microglial phagocytosis of Aβ [32] or inhibiting the uptake and degradation of Aβ by neuronal cells [56]. Tat-induced accumulation of Aβ is also observed in rat hippocampal cell cultures when recombinant Tat protein is added [57] or in the APP/PS1 mouse brain when Tat expression is regulated by an inducible system [33]. Increased Aβ deposition in the mouse brain in the presence of Tat is consistent with our observation (Figure 5*G and F*) that the number and size of Aβ plaques in the hippocampus increased in Lenti-Tat-injected brains, and with our finding that Aβ42 is dramatically increased when Tat protein is expressed in astrocytes (Figure 4*B*). These data demonstrate that HIV-1 Tat directly interacts with and modulates the trafficking and processing of APP, resulting in increased Aβ production. However, recombinant mTat (C22G) protein failed to induce Aβ production in rat hippocampal cell cultures [57], whereas the expression of mTat (K41E) in astrocytes induced a relatively small but significant increase (Figure 4*A and B*). This may be due to several reasons, including the different cell types used for the experiments and whether recombinant Tat was added to the extracellular medium or was expressed intracellularly. It is important to take into consideration the position and function of the mutated amino acid in this instance, as cysteine 22 is required for many Tat functions. For example, it is essential for the Zn^2+^ chelation required for Tat dimerization, which may be required for Tat-mediated up-regulation of Aβ production [58]. Further study will be required to fully understand the role of cysteine 22 in the Tat-dependent proteolytic processing of APP.

It can be argued that the increase in Aβ42 mediated by Tat is caused by the inhibition of neprilysin or by inhibiting the uptake of Aβ by microglial cells, rather than by the direct interaction between Tat and APP. However, our data and data from previous experiments [31], [32] indicate that the interaction of Tat with APP and subsequent modulation of APP processing is the primary cause of increased Aβ42 levels in Lenti-Tat-infected U-87 MG cells and APP/PS1 transgenic mice (Figure 4 and 5). First, Tat proteins also increased Aβ42 levels in astrocytes, thus excluding the effect of Tat on microglial cells. Second, our data showed that the amount of β-CTF, as well as Aβ42, increased in Lenti-Tat-infected U-87 MG cells, while the amount of APP was unchanged (Figure 4*A*). The level of β-CTF is unaffected by overexpression or inhibition of neprilysin [59], [60], suggesting that the increase in Aβ42 in Lenti-Tat-infected U-87 MG cells resulted from the modification of APP processing by Tat. These data suggest that the interaction between Tat and APP is a primary cause of the increase in Aβ.

The fact that the relocalization of APP (Figure 2) is dependent on the level of Tat expression supports the hypothesis of an interaction between Tat and intracellular APP. However, Tat is secreted from virus-infected cells and then taken up by surrounding cells, which requires LRP binding [8]. In addition, the addition of extracellular Tat protein increased the Aβ level in cultured rat hippocampal cells [57]. Therefore, Tat may play dual roles in Aβ production, whereby intracellular Tat interacts with APP and affects its subcellular trafficking, while extracellular Tat interacts with LRP and inhibits the uptake and degradation of Aβ. Additional work will be required to understand the relationship between Tat, APP, and LRP, as well as the mechanism of APP processing in the presence of Tat.

Although the mTat protein (K41E) did not show any differences in interaction or colocalization with APP or in the recruitment of APP into lipid rafts, it induced 1.6-fold less Aβ42 in cultured cells compared with wild-type Tat (Figure 4*B*). This is consistent with the relatively smaller amount of β-CTF produced by mTat (Figure 4*A*). As previously noted, Tat regulates the production of Aβ through various mechanisms [31], [32], [56]. The conformational change induced by the substitution of lysine for glutamic acid (K41E) might have impaired the ability of Tat to interact with neprilysin [31], inhibit microglial phagocytosis [32], or interact with LRP [56]. Lysine 41 resides in the middle of the core domain and is essential for Tat interaction with LRP; thus, the association of mTat with LRP may be inhibited by the conformational change in mTat. Therefore, the uptake and degradation of Aβ might not be effectively inhibited by mTat, resulting in lower Aβ production compared with wild-type Tat. This hypothesis requires further investigation.

In conclusion, this study identified HIV-1 Tat as a potential regulator of Aβ production through direct interaction with and translocation of APP. The work presented here provides new insights into the role of HIV-1 viral proteins in the pathogenesis of HAND in the post-HAART era.

## Methods

### Ethics statements

This study was carried out in strict accordance with the recommendations in the Guide for the Care and Use of Laboratory Animals of the Korea Food and Drug Administration. Animal studies including the issue of ethical treatment of the animals were all reviewed and approved by the Inje University Aniaml Care and Use Committee of Inje University Animal Resource Center (Permit number: 2010-52). All surgery was performed under ketamine anesthesia, and all efforts were made to minimize suffering.

### Cells

U-87 MG human glioma cells (ATCC), SK-N-MC human blastoma (Korean Cell Line Bank, Seoul, Korea), SH-SY5Y human blastoma (Korean Cell Line Bank, Seoul, Korea) and 293FT human epithelial kidney cells (Invitrogen) were cultured at 37°C in Dulbecco's modified Eagle's medium (DMEM, Hyclone Laboratories. Inc, Logan, Utah) supplemented with 10% fetal bovine serum (FBS, Gibco-BRL, Invtrogen Grand Island, N.Y.), and antibiotic-antimycotic (Gibco-BRL, Invitrogen, Grand island, N.Y.). SH-SY5Y cells were transduced with Lenti-GFP, Lenti-Tat or Lenti-mTat virus and incubated for 2 days in the presence of 2 µg/ml puromycin.

### Plasmids

pcDNA2.1-Tat HeLa cells were transfected with pNL4-3 plasmid [61] and incubated for 24 hours. Total RNA was purified from HeLa cell extracts by using TRIzol reagent (Invirogen, Scotland, UK). 5 µl of RNA was used for RT-PCR by using avian myeloblastosis virus reverse transcriptase (AMV-RT, Promega, Madison, WI) with oligo(dT) and Tat-forward primers. *tat* sequence was amplified with following primers Tat-forward; 5′-GCAGGATCCATGGAGCCAGTAGAT-3′ and Tat-reverse; 5′-GCGGCCGCCTATTCCTTCGGGCCTGTC-3′. Amplification condition was 95°C for 1 min, followed by 25 cycles of denaturation at 95°C for 1 min, annealing at 58°C for 30 s, and elongation at 72°C for 30 S. Amplified *tat* sequence was cut by BamHI and Not I, inserted into BamHI and NotI cut pcDNA3.0 plasmid (Invitrogen).

Mutant *tat* was generated by PCR amplification of pcDNA3.0-Tat with the mutant primers. First, two independent PCR reactions were performed with the two sets of primers. Set1; Tat-forward plus Tatcm2; 5′-AGATGCCTAAGGCTTCTGTTGTGAAACAAAC-3′, and set2; Tatcm1; 5′-GTTTGTTTCACAACAGAAGCCTTAGGCATCT-3′ plus Tat-reverse. PFU (BIONEER, Daejon, Korea) was used as the DNA polymerase and the same PCR condition with the wild-type tat amplification was used. The PCR product was run on a 1% agarose gel and the DNA was cut, both gel fragments were put in an 1.5 ml tube and 100 µl of DW were added. The gel slices were then incubated for 15 min at 37°C. 20 µl of DNA solution was taken from each tube, mixed and used as a template for a second PCR. In the second PCR, Tat-forward and Tat-reverse were used as a primer set. The second PCR products were run on 0.9% agarose gel and purified using the MinElute Gel Extraction kit (QIAGEN, Hilden, Germany). Purified DNA was cut with BamHI and NotI and ligated with pcDNA3.0 that is cut by BamHI and NotI (pcDNA3.0-mTat).

pGEXT4-Tat was generated by cutting the pcDNA3.0-Tat with BamHI and NotI restriction enzyme and ligating with BamHI and NotI cut pGEXT4 (GE Healthcare) plasmid. pHyk-Tat and pHyk-mTat was generated by cutting the pcDNA3.0-Tat or pcDNA3.0-mTat with BamHI and Xho I restriction enzyme and ligating it with pHyk [62] that is cut by BamHI and Xho I. Lentiviral vector expressing Tat under the control of hCMV (pLentiH1.4-Tat) was generated by cutting pcDNA3.0-Tat with BamHI and XhoI restriction enzyme and ligating it with pLentiH1.4 that is cut by BamHI and XhoI. Lentiviral vector expressing Tat under the control of synapsin promoter (pLentisyn1.4-Tat) was generated by restriction enzyme digestion of pHyk-Tat with BamHI and XhoI and insertion into BamHI and Xho I digested pLentisyn1.4 vector. Both pLentiH1.4 and pLentisyn1.4 was generated by modification of pNL4-3 in the lab. pCB6-APP [63], the plasmid endcoding C-terminally Myc-tagged APP was a kind gift from Dr. Mook I-H, Seoul National Univeristy (Seoul, Korea)

### Antibodies

Antibody 22C11 against aa 66–81 of the APP N-terminus was purchased from Millipore (MAB348, EMD Millipore corporation, MA). Antibody raised against aa 676–695 of the APP C-terminus was purchased from Sigma (A8717, Sigma-Aldrich, Saint Louis, Missouri). Antibody 6E10 that recognizes aa 1–17 of Aβ was purchased from Abcam (ab12266, Abcam, Cambridge, UK). Anti-Tat antibody was produced from a hybridoma cell (1D9, cat. no. 7373) was kindly provided by the NIH AIDS Research and Reference Reagent program. Antibodies to c-Myc (Santa Cruz Biotechnology Inc. Santa Cruz, California), flotillin-1 (ab41927, Abcam, Cambridge, UK), MAP2 (ab32454, Abcam, Cambridge, UK), transferrin receptor (LS-B6156, LSBio) and actin (Sigma-Aldrich, Saint Louis, Missouri) were purchased from the manufacturer. Fluorochrome-conjugated antibodies were purchased from Jackson Immunoresearch Laboratories (Jackson Immunoresearch Laboratories Inc., West Grove, PA).

### GST-Pulldown Assay

pGEX4T or pGEX4T-Tat were transformed into *E. Coli* BL21 strain. 1 mM isopropyl-1-thio-galactosidase (IPTG) was added to each E.coli cell culture at OD600 = 0.6 to induce to expression of GST or GST-Tat and incubated further 3 hours. Cells were harvested by centrifugation at 6500 rpm, 4°C for 15 min and the pellet was resuspended in EBC buffer (50 mM Tris. Cl pH 8.8, 120 mM NaCl, 0.5% Nodient p-40 (NP-40)) supplemented with protease inhibitors (cOmplete Mini, Roche) and 2 mM 1,1-Dithiothreitol (DTT). Cells were sonicated and the supernatant was separated by centrifugation at 13,000 rpm, 4°C for 15 min. 10 µl of glutathione-sepharose bead (Peptron, Korea) was added to the supernatant and incubated for 12 hours at 4°C. Beads were washed with EBC buffer supplemented with 2 mM DTT and 0.075% sodium dodecyl sulfate (SDS) twice. SK-N-MC cells were harvested and resuspended in phosphate buffered saline (PBS) supplemented with 1% NP-40 and protease inhibitors. Cells were sonicated briefly and centrifuged at 13,000 rpm, 4°C for 15 min. One milligram of cell extracts was applied to GST or GST-Tat beads and incubated for 3 hours at 4°C. Beads were washed three times with PBS, bound proteins were eluted by boiling in SDS-PAGE buffer and analyzed by 8% SDS-PAGE followed by western blotting with anti-APP (22C11) antibody.

Purified recombinant APP (cat. no APP-526H, Creative BioMart, NY, USA) was resuspended in PBS supplemented with 1% NP-40 to yield a final concentration of 1 ng/µl, and 500 µl was incubated with GST- or GST-Tat-coated beads for 3 hours at 4°C. The beads were washed three times with PBS supplemented with 1% NP-40. The bound proteins were eluted by boiling in SDS-PAGE buffer, separated in 8% SDS-PAGE gels, and analyzed by western blotting with an anti-APP (A8717) antibody.

To examine the affinity of Tat for APP, SK-N-MC cells were resuspended in PBS supplemented with 1% NP-40 and briefly sonicated. The extracts were applied to GST- or GST-Tat-coated beads and incubated for 3 hours at 4°C. The GST-coated beads were washed three times with PBS supplemented with 1% NP-40. The GST-Tat-coated beads were then washed three times with PBS containing 137 (PBS), 200, 300, 400 or 500 mM NaCl and 1% NP-40. Bound proteins were eluted by boiling in SDS-PAGE buffer and then analyzed by separation in 8% SDS-PAGE gels followed by western blotting with an anti-APP (A8717) antibody.

### Immunoprecipitation

6×10^5^ of HEK 293FT cells were plated in 6-well plate. Cells were transfected with pHyk-Tat plasmid alone or in combination with pCB6-APP using Lipofectamine (Invitrogen) and incubated for 48 hours. 3×10^5^ of U-87 MG cells in 6-well plate were transduced with Lenti-Tat, Lenti-mTat or mock virus and incubated for 48 hours. Control or Tat-expressing cells were resuspened in PBS containing 1% NP-40 and protease inhibitors and incubated for 30 min at 4°C with rotation. Supernatant was separated by centrifugation at 12,000 rpm, 4°C for 30 min and anti-Tat (7383), anti-c-Myc (9E10, sc-40, Santa Cruz) or anti-APP (ab12266, Abcam) antibody was added and incubated for 1 h on ice. 10 µl of protein A-Sepharose CL4B (Amersham Pharmacia Biosciences) was added and further incubated overnight at 4°C with rotation. Protein A beads were pelleted by centrifugation at 1,000 rpm, 4°C for 5 min and washed three times with PBS. Bound proteins were eluted by boiling with SDS-loading dye and separated on SDS-PAGE followed by western blotting with anti-Tat, anti-APP (22C11), or anti-c-Myc antibody.

### Western blotting

Samples were subjected to SDS-PAGE and transferred to polyvinylidene difluoride (PVDF) membrane (BIO-RAD, Hercules, CA) by electroblotting. The antibodies used were anti-APP (MAB348), anti-c-Myc (sc-40), anti-Tat, anti-actin, anti-CTF (ab12266 and A8717), anti-flotillin-1 (ab41927) and anti-Aβ (ab12266) antibodies. The secondary antibodies were goat anti-mouse (172–1011 BIO-RAD, Hercules, CA) and donkey anti-rabbit (NA934, Aemrsham-Pharmiacia Biosciences) antibody conjugated with horseradish peroxidase (HRP). Chemiluminescent signals were detected by ECL western blot detection reagents (GE Health care, Buckinghamshire, UK).

### Preparation of detergent-soluble and detergent-insoluble fractions

Fractions were separated as described previously with minor modification [64]. U-87 MG cells were transduced with mock- or Lenti-Tat virus and incubated for 20 days. Cells were split and counted, and equal numbers of mock- or Tat-virus infected U-87 MG cells (1×10^6^) were lysed in 10 mM PIPES (pH 6.7), 50 mM KCl, 1 mM MgCl_2_, 2M glycerol, 0.5% Triton X-100. After incubation for 15 min at 4°C, the cell extracts were centrifuged at 12,000 rpm at room temperature for 10 min. The supernatant was taken as the detergent-soluble fraction. Pellets were resuspended in 50 mM Tris. Cl pH 7.5, 10 mM EDTA, 100 mM NaCl, 1% SDS, homogenized by passing 10 times through 25 gauge needle and boiled for 10 min. extracts were centrifuged at room temperature at 12,000 rpm for 10 min, supernatant was removed and referred to as the detergent insoluble fraction. Proteins at each fractions were analyzed by 10% SDS-PAGE followed by western blotting with anti-APP (A8717) antibody.

### Immunofluoresence

U-87 MG cells were seeded onto circular glass coverslip (18 mm, Marlenfeld, Lauda-Konlgshofen, Germany) in 12-well plate and incubated for 24 hours. Cells were transfected with the expression construct for Tat (pHyk-Tat or pHyk-mTat) using Lipofectamine (Invirogen). After 16 h incubation, the cells were fixed for 10 min with 4% paraformaldehyde, permeabilized with 0.1% Triton X-100 for 5 min, and blocked with 4% BSA for 1 h. Cells were then incubated for 2 h with anti-Tat antibody (1∶200 dilution) followed by FITC-conjugated anti-mouse antibody (1∶200 dilution). Subsequently, they were treated for 2 h with anti-APP (A8717, 1∶1000 dilution) or followed by rhodamine-conjugated anti-rabbit antibody (1∶200 dilution), and washed thoroughly with PBS. Cells were visualized using an inverted phase fluorescence microscope (Carl Zeiss, Axiovert 200 with Axiovision software). Brain sections were stained by free-floating method as described previously [65]. Mouse brain was perfused and cryoprotected by immersion in 30% sucrose followed by cryosectioning in the sagital plane at 35 µm. The brain slices were fixed for 15 min with 4% paraformaldehyde in PBS and washed three times with PBS for 5 min followed by permeabilization for 20 min with 0.5% Triton X-100 in PBS. Sections were washed three times with PBS and incubated for 1 h in 5% normal goat serum (sc-2043, Santa Cruz), 3% bovine serum albumin, and 0.1% Triton X-100. Anti-Tat (7383) antibody (1∶100 dilution) was added and incubated for 2 h at room temperature followed by FITC-conjugated anti-mouse antibody (1∶200 dilution). Subsequently, sections were treated with anti-MAP2 (ab32456, Abcam, 1∶200 dilution) antibody followed by rhodimine-conjugated anti-rabbit antibody (1∶200 dilution). After washing, sections were mounted on slide glass and air-dried for 20 min. A drop of fluorescence mounting medium (Dako, S3023) was added and coverslipped. Sections were visualized using an inverted phase fluorescence microscope (Carl Zeiss, Axiovert 200 with Axiovision software).

### Immunohistochemistry

Mouse brain was perfused and cryoprotected by immersion in 30% sucrose followed by cryosectioning in the sagital plane at 35 µm. The brain slices were fixed for 15 min with 4% paraformaldehyde and permeabilized for 20 min with 0.5% Triton X-100. Antigen was retrieved by incubating the brain sections in 70% formic acid for 10 min followed by incubation for 15 min in 0.3% hydrogen peroxide. Sections were incubated for 1 h in 5% normal goat serum, 3% bovine serum albumin, and 0.1% Triton X-100. Anti-Aβ antibody (6E10, ab12266, 1∶50 dilution) was added to the slices and incubated overnight. After washing with PBS, slices were developed using the DAB kit according to the manufacturer's instructions (Dako Real, Dako). After washing with PBS, sections were mounted on slide glass and air-dried. A drop of histochemical mounting medium (National diagnostics, Atlanta, Georgia) was added and coverslipped. Sections were visualized using an inverted phase fluorescence microscope (Carl Zeiss, Axiovert 200 with Axiovision software).

### ELISA

Mouse brain was perfused, and the hippocampus was dissected and homogenized in buffer A (50 mM TrisCl pH 7.5, 150 mM NaCl, 0.1% Triton X-100). After ultracentrifugation at 100,000× g for 1 h at 4°C, the supernatant was collected as the soluble fractions. 1.5×10^5^ of U-87 MG cells in 12-well plate were transduced with 400 µl of mock, Lenti-mTat or Lenti-Tat virus. Virus-infected U-87 MG cells were cultured for 9 days and the conditioned media were collected. The cell numbers were counted and used for the calculation of Aβ42 concentration. The ELISA was performed according to the manufacturer's instructions (Cat No. 27710 Immunobiological Laboratories).

### Stereotaxic Injection

Lentiviruses were stereotaxically injected into the hippocampi of 2-month-old APP/PS1 mice as described previously [66]. Mice were anesthetized with an intraperitoneal injection of a mixture of ketamine (100 mg/kg) and xylazine (10 mg/kg) and placed in a stereotaxic head holder (Stoelting Co., Wood Dale, Illinois). An incision was made on the middle of the scalp and a hole was surgically drilled through the skull above the injection site. Virus (1 µl) was injected using a 10 µl Hamilton syringe with a 28 gauge needle at a speed of 0.2 µl/min with an automatic injector (Stoelting Co.). The needle was left in place for an additional 10 min before withdraw. Stereotaxic injections were delivered to the hippocampus at the following coordinates (in mm): anterior −1.8, lateral ±1.3, and ventral −1.8. The anterior and lateral coordinates were calculated from the bregma, and the ventral coordinates were calculated from the skull surface. After injection, the needle was removed and the scalp was closed with suture.

### Lentivirus Production and Concentration

Lentivirus was produced as described previously with some modification [67]. Wild-type Tat- or mTat-expressing constructs under the control of the synapsin or hCMV promoter were cloned into the lentiviral vector. Lentiviral vector containing only synapsin or hCMV promoter were cloned, virus particles were produced, and used as control virus (Mock). 6×10^5^ of HEK 293FT [68] cells were seeded in 6 well plate 24 h prior to transfection. 0.48 µg of transfer vector, 0.48 µg of packaging plasmid (psPax2) and 0.24 µug of envelop plasmid (pMD2.G [69]) were transfected into HEK 293FT cells with Lipofectamine Reagent (Invitrogen) and incubated for 48 h. The supernatant was harvested, cleared by centrifugation at 1,500 rpm for 5 min. and filtered through 0.22 µM cellulose acetate filter (Sartorius Stedim biotech, Goettingen, Germany). For the stereotaxic injection, virus was concentrated by ultrafiltration and ultracentrifugation. Filtered virus supernatant was applied onto Amicon-20 columns (EMD Millipore Corporation, Billerica, MA) and centrifuged at 1,500 rpm, 4°C. Concentrated virus was overlaid on 2 ml of 20% sucrose in PBS and ultracentrifuged at 40,000 rpm for 2 h 30 min at 4°C in the CS 120GXL (Hitachi, Japan) micro ultracentrifuge with S52ST swing rotor. Supernatants were removed and the virus particles were resuspended in PBS.

### Lipid Raft Isolation

Lipid rafts were isolated from mock-, Lenti-mTat or Lenti-Tat infected U-87 MG cells as described previously [70]. 3×10^5^ of U-87 MG cells were seeded in 6-well plate 24 h prior to virus infection. 800 µl of virus supernatant with 8 µg/ml of polybrene were applied to the cells and incubated for 8 h. Virus supernatant were removed, fresh media were added and the cells were further cultured for 14 days. Virus-infected U-87 MG cells were harvested, resuspended in 400 µl of MES-buffered saline (MBS: 25 mM MES pH 6.5, 150 mM NaCl, 1% Triton X-100), and disrupted by passing the suspension through a 25-gauge needle 10 times. An equal volume of 80% sucrose in MBS was added and the mixture was placed in the bottom of 5-ml ultracentrifuge tube. MBS containing 35% and 5% sucrose was successively laid over the cell extracts and the sucrose gradients were centrifuged at 100,000× g for 16 h at 4°C in the CS 120GXL (Hitachi, Japan) micro ultracentrifuge with S52ST swing rotor. Fractions were collected in 0.5 ml volumes from the top to the bottom of the tube. The localization of APP proteins as well as flotillin-1, transferrin receptor and Tat was examined by 10% (APP, transferrin receptor and flotillin-1) or 15% (Tat) SDS-PAGE followed by western blotting.

## Supporting Information

Figure S1 HIV-1 Tat associate with APP. (A) A Tat mutant form, mTat (TatK41E), is deficient transactivation activity. Plasmid pHyk (control), pHyk-Tat, or pHyk-mTat was transfected into Magi (HeLa-CD4-LTR-β-gal) cells and incubated for 2 days. X-gal staining of transfected cells clearly showed that mTat does not transactivate the HIV-1 LTR, whereas wild-type Tat exhibited strong transactivation activity (*B*) Recombinant purified APP. 200 ng of recombinant purified APP was run on 8% SDS-PAGE and stained with commassie brilliant blue. (*C*) Association of recombinant APP with Tat. Recombinant APP proteins were applied to GST or GST-Tat beads and the association with Tat was examined by western blotting with an antibody against APP (A8717). 80 ng of recombinant APP was loaded for loading control. (*D*) Purified GST-Tat deletion mutants. GST and GST-Tat mutants were purified by glutathione-sepharose bead. Bound proteins were boiled and run on 12% SDS-PAGE and stained with commassie brilliant blue. (*E*) Purified GST-Tat mutants. GST-Tat mutants were purified by glutathione-sepharose bead. Bound proteins were boiled and run on 12% SDS-PAGE and stained with commassie brilliant blue. (*F*) Association of Tat mutant with APP. GST pulldown assay with SK-N-MC neuroblastoma cell lysates shows that mutant Tat proteins interact with APP.
(TIF)

Figure S2 Fluorescence microscopy image of pEYFP-Tat-transfected U-87 MG cells. (*A*)U-87 MG cells were transfected with pEYFP-Tat and incubated for 24 h. Picture was taken without fixation. Scale bar = 100 µm. (*B*) Fluorescence microscopy images of mTat-transfected U-87 MG cells immunostained with anti-Tat and anti-APP (A8717) antibodies are shown. U-87 MG cells were transfected with the wild-type Tat construct and incubated for 16 h. The cells were fixed and stained with anti-Tat or anti-APP antibodies followed by FITC-conjugated anti-mouse or rhodamine-conjugated anti-rabbit antibodies, respectively. Scale bar = 10 µm.
(TIF)

Figure S3 Large amounts of APP were moved to lipid raft fraction in Lenti-mTat -infected U-87 MG cells. U-87 MG cells were transduced with Lenti-mTat virus and incubated for 14 days. Cells were harvested and lysed in the presence of 1% Triton X-100 and subjected to 5% and 35% discontinuous sucrose density gradient ultracentrifugation. Fractions of 0.5 ml were harvested from the top to the bottom and analyzed by Western blotting for APP, flotillin-1, and Tat.
(TIF)

Figure S4 The Tat protein shows cytotoxicity in SH-SY5Y cells. SH-SY5Y cells were transduced with Lenti-GFP, Lenti-Tat, or Lenti-mTat and incubated for 3 days in the presence of 2 µg/ml puromycin. mTat protein shows attenuated cytotoxicity compared with Tat.
(TIF)

Figure S5 HIV-1 Tat increases the levels of Aβ42. (*A*) Lenti-Tat-infected cells show retarded growth. Mock, Lenti-Tat, or Lenti-mTat were transduced into U-87 MG cells. Cells were incubated for 12 days. The cell number was counted every 3 days. Lenti-Tat-infected cells showed greatly retarded growth, whereas Lenti-mTat-infected cells showed growth similar to mock-infected U-87 MG cells. (*B*) Total Aβ42 peptide produced from virus-infected cells. Conditioned medium was harvested from cells infected with each virus and used to detect Aβ42 by ELISA. (*C*) The concentration of Aβ42 produced by mock-, Lenti-Tat-, or Lenti-mTat-infected U-87 MG cells. The concentration was calculated as the amount of Aβ42 produced by 1×10^5^ cells.
(TIF)

Figure S6 Tat protein was primarily expressed in neuronal cells in the APP/PS1 mouse hippocampus. Lenti-Tat injected mice were sacrificed at 2 months after injection and the brains were frozen and sectioned for immunostaining with anti-Tat and anti-MAP2 antibodies. Tat protein was expressed throughout the hippocampus, including CA1 (*A*) and the dentate gyrus (DG) (*B*). Tat colocalized with MAP2 in CA1 (*A*), CA2, CA3, and the DG (*B*) of the hippocampus. Scale bar in A = 100 µm; Scale bar in B = 10 µm.
(TIF)
