# Supplementary figures and images for: HIV-1 Tat Interacts with and Regulates the Localization and Processing of Amyloid Precursor Protein
Source: PLoS One. 2013 Nov 29;8(11):e77972. doi: 10.1371/journal.pone.0077972 (PMC3843664; doi:10.1371/journal.pone.0077972)

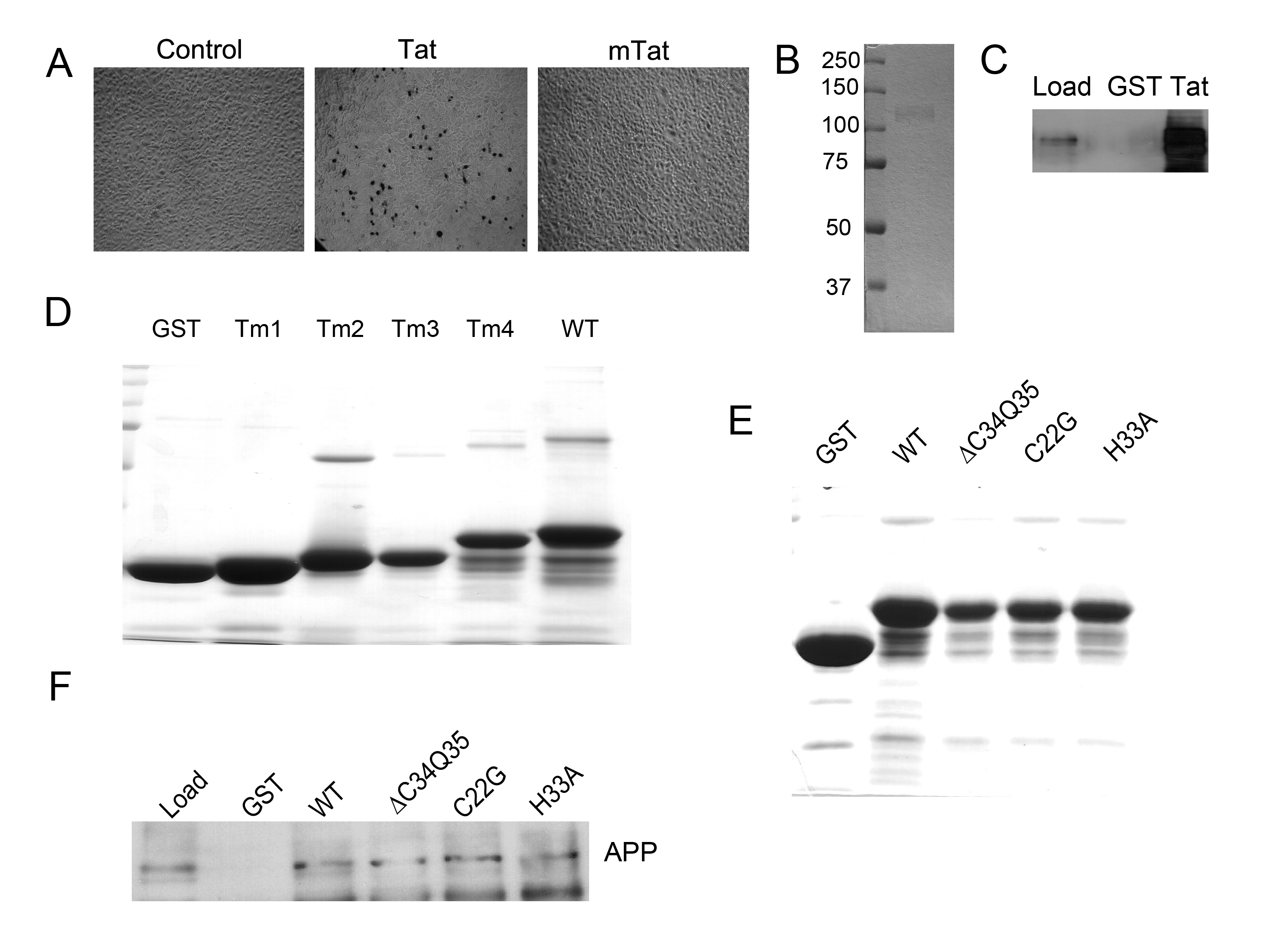

Supplement: Figure S1 — HIV-1 Tat associate with APP. (A) A Tat mutant form, mTat (TatK41E), is deficient transactivation activity. Plasmid pHyk (control), pHyk-Tat, or pHyk-mTat was transfected into Magi (HeLa-CD4-LTR-β-gal) cells and incubated for 2 days. X-gal staining of transfected cells clearly showed that mTat does not transactivate the HIV-1 LTR, whereas wild-type Tat exhibited strong transactivation activity (B) Recombinant purified APP. 200 ng of recombinant purified APP was run on 8% SDS-PAGE and stained with commassie brilliant blue. (C) Association of recombinant APP with Tat. Recombinant APP proteins were applied to GST or GST-Tat beads and the association with Tat was examined by western blotting with an antibody against APP (A8717). 80 ng of recombinant APP was loaded for loading control. (D) Purified GST-Tat deletion mutants. GST and GST-Tat mutants were purified by glutathione-sepharose bead. Bound proteins were boiled and run on 12% SDS-PAGE and stained with commassie brilliant blue. (E) Purified GST-Tat mutants. GST-Tat mutants were purified by glutathione-sepharose bead. Bound proteins were boiled and run on 12% SDS-PAGE and stained with commassie brilliant blue. (F) Association of Tat mutant with APP. GST pulldown assay with SK-N-MC neuroblastoma cell lysates shows that mutant Tat proteins interact with APP. (TIF) [file pone.0077972.s001.tif]

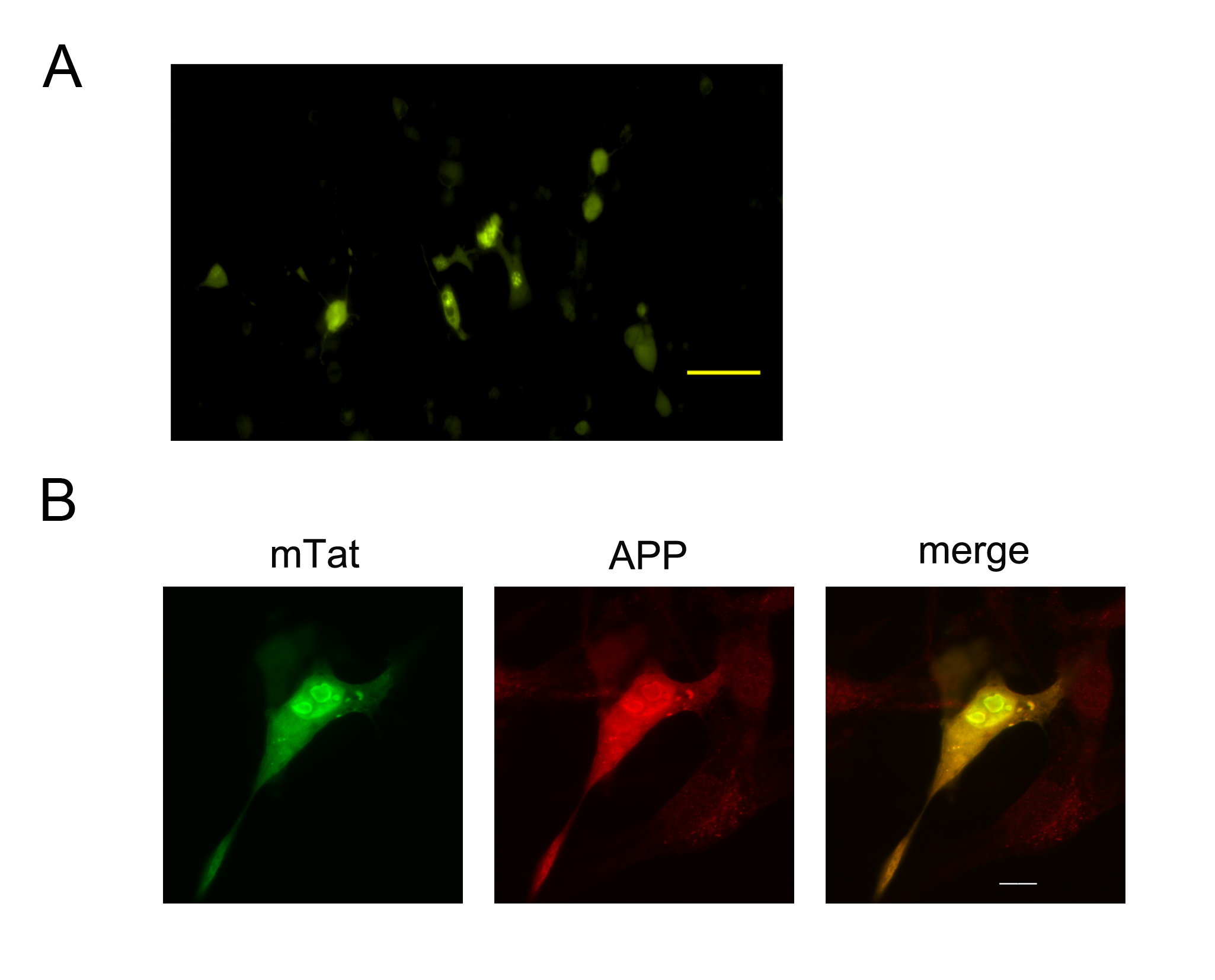

Supplement: Figure S2 — Fluorescence microscopy image of pEYFP-Tat-transfected U-87 MG cells. (A)U-87 MG cells were transfected with pEYFP-Tat and incubated for 24 h. Picture was taken without fixation. Scale bar = 100 µm. (B) Fluorescence microscopy images of mTat-transfected U-87 MG cells immunostained with anti-Tat and anti-APP (A8717) antibodies are shown. U-87 MG cells were transfected with the wild-type Tat construct and incubated for 16 h. The cells were fixed and stained with anti-Tat or anti-APP antibodies followed by FITC-conjugated anti-mouse or rhodamine-conjugated anti-rabbit antibodies, respectively. Scale bar = 10 µm. (TIF) [file pone.0077972.s002.tif]

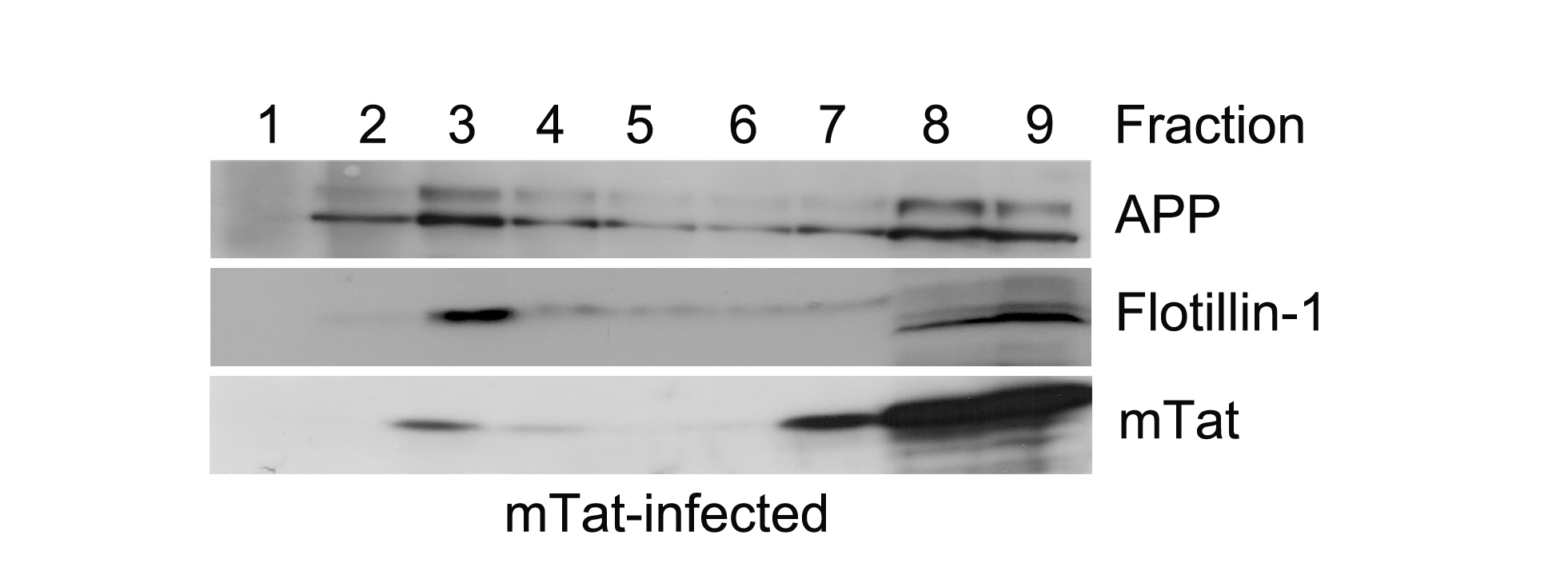

Supplement: Figure S3 — Large amounts of APP were moved to lipid raft fraction in Lenti-mTat -infected U-87 MG cells. U-87 MG cells were transduced with Lenti-mTat virus and incubated for 14 days. Cells were harvested and lysed in the presence of 1% Triton X-100 and subjected to 5% and 35% discontinuous sucrose density gradient ultracentrifugation. Fractions of 0.5 ml were harvested from the top to the bottom and analyzed by Western blotting for APP, flotillin-1, and Tat. (TIF) [file pone.0077972.s003.tif]

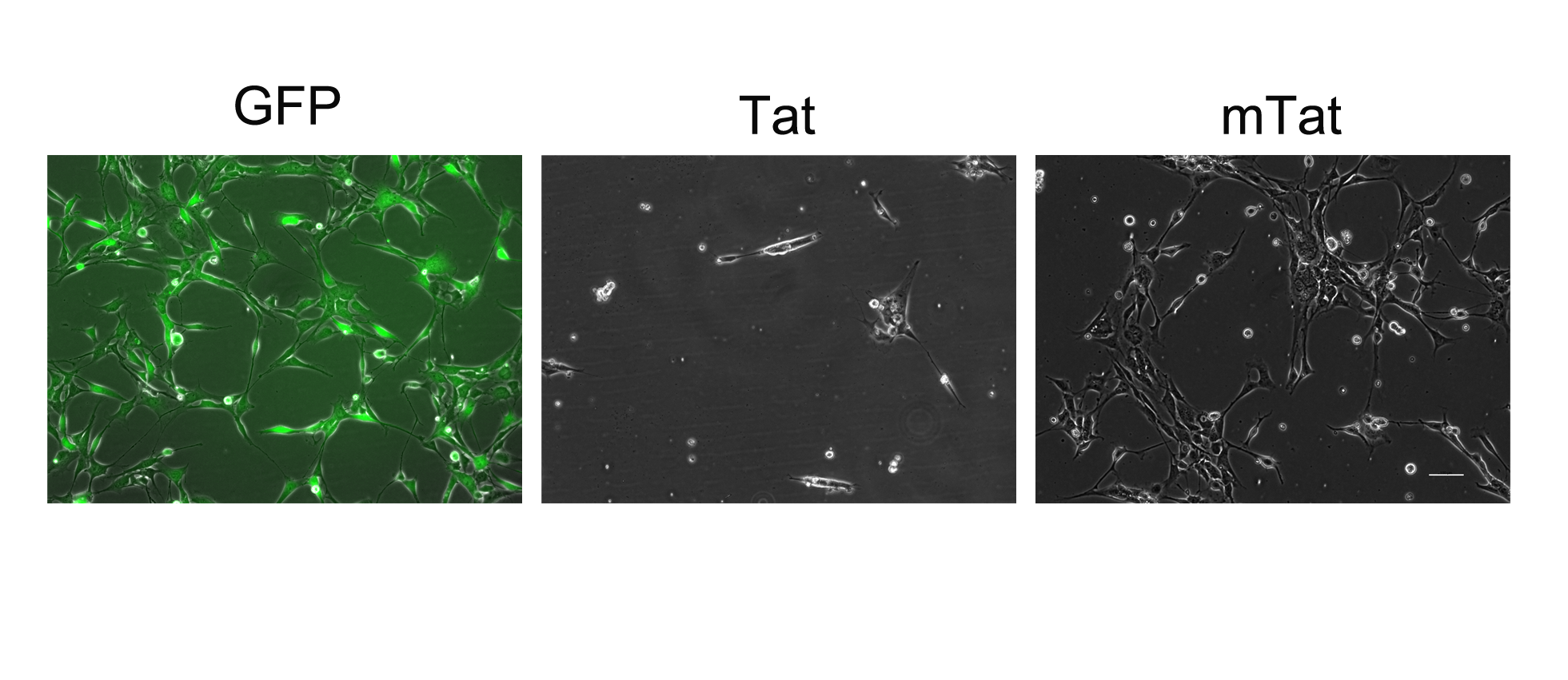

Supplement: Figure S4 — The Tat protein shows cytotoxicity in SH-SY5Y cells. SH-SY5Y cells were transduced with Lenti-GFP, Lenti-Tat, or Lenti-mTat and incubated for 3 days in the presence of 2 µg/ml puromycin. mTat protein shows attenuated cytotoxicity compared with Tat. (TIF) [file pone.0077972.s004.tif]

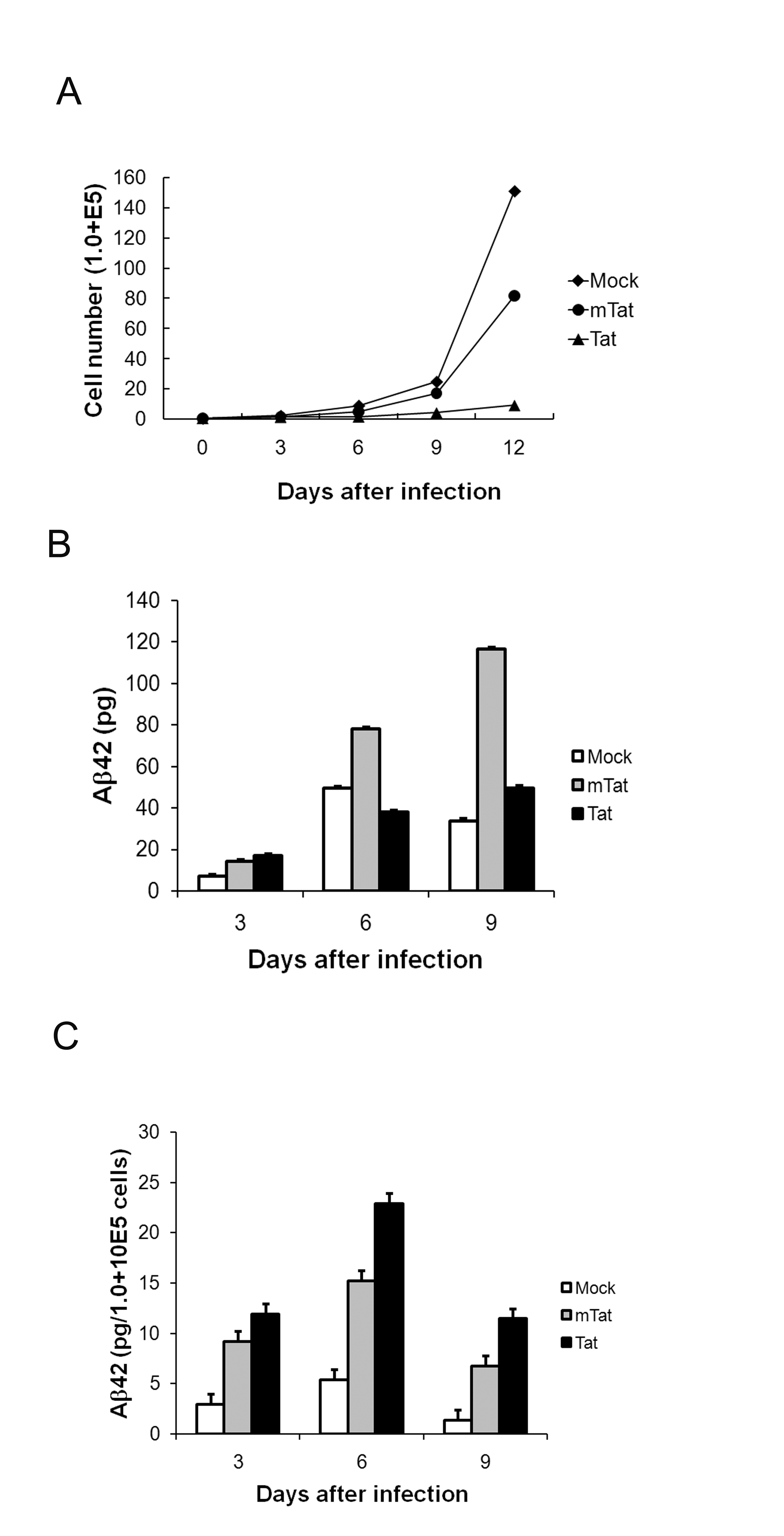

Supplement: Figure S5 — HIV-1 Tat increases the levels of Aβ42. (A) Lenti-Tat-infected cells show retarded growth. Mock, Lenti-Tat, or Lenti-mTat were transduced into U-87 MG cells. Cells were incubated for 12 days. The cell number was counted every 3 days. Lenti-Tat-infected cells showed greatly retarded growth, whereas Lenti-mTat-infected cells showed growth similar to mock-infected U-87 MG cells. (B) Total Aβ42 peptide produced from virus-infected cells. Conditioned medium was harvested from cells infected with each virus and used to detect Aβ42 by ELISA. (C) The concentration of Aβ42 produced by mock-, Lenti-Tat-, or Lenti-mTat-infected U-87 MG cells. The concentration was calculated as the amount of Aβ42 produced by 1×10^5^ cells. (TIF) [file pone.0077972.s005.tif]

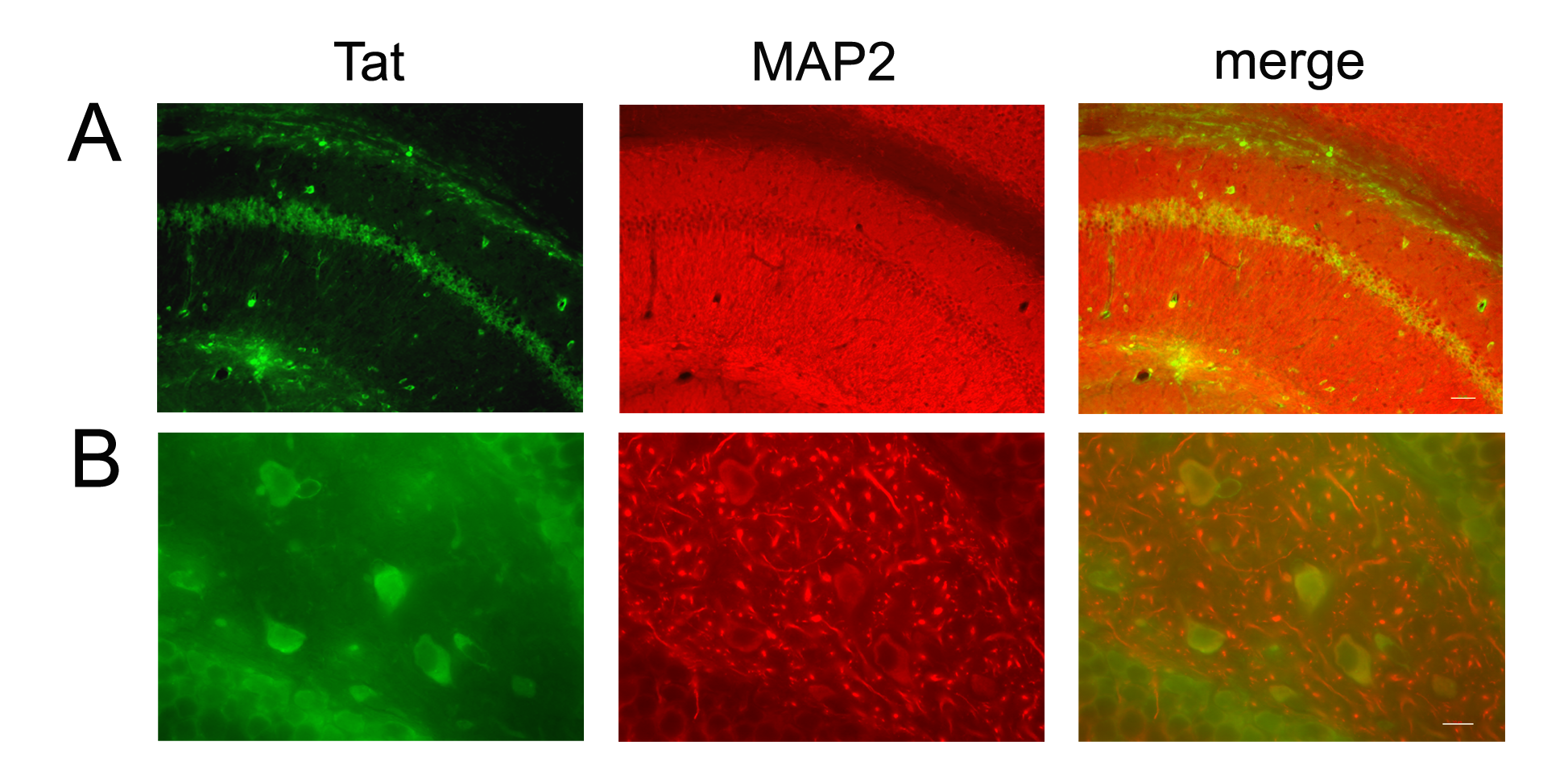

Supplement: Figure S6 — Tat protein was primarily expressed in neuronal cells in the APP/PS1 mouse hippocampus. Lenti-Tat injected mice were sacrificed at 2 months after injection and the brains were frozen and sectioned for immunostaining with anti-Tat and anti-MAP2 antibodies. Tat protein was expressed throughout the hippocampus, including CA1 (A) and the dentate gyrus (DG) (B). Tat colocalized with MAP2 in CA1 (A), CA2, CA3, and the DG (B) of the hippocampus. Scale bar in A = 100 µm; Scale bar in B = 10 µm. (TIF) [file pone.0077972.s006.tif]
